# Cloth Tents

**Published:** 1875-10

**Authors:** C. Henri Leonard

**Affiliations:** Detroit, Mich.


### CLOTH TENTS.

By C. HENRI LEONARD, M.D., Detroit, Mich.

I have been using quite extensively in my gynecological practice, a tent that I have made out of cloth. It has proven of so much service to me that I have thought it might be of value to give it to the profession.

A "cloth tent" is not a new thing *in toto;* for Dr. V. A. Taliafero, of Columbus, Georgia, wrote of them some three years ago.* To him the introduction of cloth tents is generally ascribed; but they were in use a great many centuries before his time, though for a little different purpose. Hippocrates† used them for anal fistulæ, after they were medicated, and, judging from the knowledge he had of the *local* treatment of female diseases, it would not be unfair to suppose that a somewhat similar use was made of them, as Taliafero has proposed. Another way Hippocrates had of making them, was to wind a *horse hair* around four or five pieces of lint laid lengthwise, till he got the proper conoidal or tent-like form.

I had used them, as Taliafero made them, for intrauterine applications, with but partial success. They were quite troublesome, from their great "slimsiness" when wet with a solution that was to be carried to the fundus, or by cervical mucus.

* The *Journal of the Gynecological Society of Boston*, 1872, p. 27.
† On *Fistulæ.*

Some time ago I had occasion to make Emmet's operation upon a conoid cervix, for the relief of dysmenorrhœa and sterility, and I made use of them for intra-cervical packing; that is, to keep the cut edges of the cervical canal from uniting. At the first application they were very readily introduced (a sponge-tent having previously been used), and seemed to be just the thing. On the next day's visit, the neck having contracted somewhat, I was completely foiled in their introduction, and so had to resort to lint plugging. It at once struck me that if a *wire* could be introduced, in some way, *into* the cloth cone (Hippocrates had stiffened them by wrapping them around with horse hair), their objectionable feature would be entirely removed.

On my return to the office, I tried rolling a piece of hair wire into the cone and found that it answered exactly. Since then I have made use of them for almost all purposes as a dressing-applicator to the uterine cavity. They are equally applicable for dressing any other sinus-like canal, whether from wounds or otherwise.

To make one, you need but a strip of linen 6 inches in length, by ¾ of an inch in width, a piece of hair wire 4 inches long, and a few inches of common thread. Roll one corner of the linen strip lightly between the thumb and finger, then unroll and place the centre of the wire at the corner so rolled, and then roll the cloth at this corner over it (*spirally*, just as you would go to work to make a paper lamp-lighter,) till you get *almost* to the other corner of the same end, then bend the wire upon itself (double it, in other words,) so that the two extremities will point to the unwound portion of the linen; this done, continue rolling the linen, in a *spiral* manner, about the doubled wire till exhausted, then tie with the thread the last spiral turn about the wire. You now have a tent about 2½ inches in length, and one sufficiently firm to enter *any* normal uterine canal, and most any abnormal one. You can bend it to any curve you choose to facilitate its introduction. It has still another advantage over all other tents, in that you can leave it *in sitû* (as I frequently do, for 24 hours,) with *no* danger to your patient, as it is *inexpansible*, and hence no excitor of metritis, though a stimulator (from its very slight mechanical irritation) to the endometrium. By so doing you can get a *prolonged* action of a medicament upon the lining membrane of the uterus, which is impossible to get by any other method of application. Further, you need not use such energetic local applications, and you may be sure that they reach the *whole* uterine cavity; something you cannot do with our intra-uterine applicators, unless you are a very skillful manipulator. The shape of the fundus-cavity is an anatomical proof of the great difficulty of making a complete application with the common metal applicators; whereas the cloth tent, by meeting with resistance at the fundus, immediately doubles upon itself, thus occupying the whole cavity.

You can make them of any *size*, and of any degree of stiffness, by increasing the thickness of cloth, and the size, or number of doublings, of your wire. I have them of all sizes, from those suitable for an ante-puberic uterus, to one as large as your index finger.

I use them now for cleansing the uterine tract previous to an application of astringents or other medicaments thereto, and find they clean away the tenacious mucus much better than a syringe or a wisp of cotton, on Emmet's applicator. Indeed, it is invaluable in many ways. By leaving the thread without the vulva, the patient can as easily and safely remove it at her residence, as can her physician. You have only to remember to tie a string (or a colored thread) to the cotton pledget you leave in the vagina, so that she may be made aware which to remove (pull) first.

Taking all these points into consideration, with their plea of cheapness and cleanliness (to the operator, for they are thrown away as fast as used, and need be touched only with the tip of the dressing forceps,) I am sure they will commend themselves to any one who will take the trouble to make up a half-dozen for trial.

I now submit a brief chronological *résumé* of "uterine tenting." In this history, as in that of all other histories, it is impossible not to recognize that *resurgam* is the epitaph of all things, and that history of the succession of ages is but the unfolding of the truthfulness of these prophecies. In other words, that a modern inventor can hope but to be a reviver of some long forgotten principle ; at least this is oftener the case than otherwise.

SPONGE TENTS.—Re-introduced by Simpson, in 1844; were known to Aetius and Hippocrates, and in constant use by them, though in the succeeding centuries overlooked and forgotten. Aetius also used *metal* uterine dilators, similar to Peaslee's of to day.

SLIPPERY ELM TENTS.—If I should say *wood* tents, then I must say *re*-introduced by Storer, of Boston, in 1855, when in Scotland, and a student of the great Simpson. It was in an article read before the Medical and Chirurgical Society of Edinburgh, that he spoke of their usefulness. Byford, in his work on the Uterus, (1870), was, probably, most instrumental in getting them before Western practitioners; though, if I recollect aright, he has given no credit to Storer, in that work. The Hippocratic gynecologists made use of hollow *wooden* tents for opening the uterus, as well as *leaden* pipelets. There was a two-fold action, in case of the wooden ones.: 1st, a swelling of the tube ; and 2nd, an opportunity for the introduction of aromatic fumes (a favorite method for treating sterility, from the shutting up of the os uteri,) into the uterine cavity. After the mouth of the womb was open they applied cantharides, myrrh, etc., to the cavity of the organ. Intra-uterine suppositories were also in vogue.

LAMINARIA DIGITATA.—Introduced in 1862, by Sloan, of Ayr, Scotland. They were then unperforated. Greenhalgh, of London, afterward conceived the idea of increasing their utility by longitudinal perforation, as we now find them in the market.

Laminaria and Sponge Combined.—I have never seen one of this kind, but Dr. Martin, of Boston (1870), speaks of their advantage over either the "laminaria" or "sponge," when they are used separately. How his combination is made, I do not know.

Cloth.—Hippocratic in their origin. Re-introduced by Dr. Taliafero, in 1872.

